# Classification of *Myoviridae *bacteriophages using protein sequence similarity

**DOI:** 10.1186/1471-2180-9-224

**Published:** 2009-10-26

**Authors:** Rob Lavigne, Paul Darius, Elizabeth J Summer, Donald Seto, Padmanabhan Mahadevan, Anders S Nilsson, Hans W Ackermann, Andrew M Kropinski

**Affiliations:** 1 Biosystems Department, Katholieke Universiteit Leuven, Kasteelpark Arenberg 21, Leuven, B-3001, Belgium; 2 Center for Phage Therapeutics, Department of Biochemistry and Biophysics, Texas A&M University, College Station, TX 77843, USA; 3 Department of Bioinformatics and Computational Biology, George Mason University, Manassas, VA20110, USA; 4 Department of Genetics, Microbiology and Toxicology, Stockholm University, S-106 91 Stockholm, Sweden; 5 Felix d'Herelle Reference Center for Bacterial Viruses, Department of Medical Biology, Faculty of Medicine, Laval University, Quebec, QC, G1K 4C6, Canada; 6 Laboratory for Foodborne Zoonoses, Public Health Agency of Canada, 110 Stone Road West, Guelph, ON, N1G 3W4, Canada; 7 Department of Molecular & Cellular Biology, University of Guelph, Guelph, ON, N1G 2W1, Canada

## Abstract

**Background:**

We advocate unifying classical and genomic classification of bacteriophages by integration of proteomic data and physicochemical parameters. Our previous application of this approach to the entirely sequenced members of the *Podoviridae *fully supported the current phage classification of the International Committee on Taxonomy of Viruses (ICTV). It appears that horizontal gene transfer generally does not totally obliterate evolutionary relationships between phages.

**Results:**

CoreGenes/CoreExtractor proteome comparison techniques applied to 102 *Myoviridae *suggest the establishment of three subfamilies (*Peduovirinae*, *Teequatrovirinae*, the *Spounavirinae*) and eight new independent genera (Bcep781, BcepMu, FelixO1, HAP1, Bzx1, PB1, phiCD119, and phiKZ-like viruses). The *Peduovirinae *subfamily, derived from the P2-related phages, is composed of two distinct genera: the "P2-like viruses", and the "HP1-like viruses". At present, the more complex *Teequatrovirinae *subfamily has two genera, the "T4-like" and "KVP40-like viruses". In the genus "T4-like viruses" proper, four groups sharing >70% proteins are distinguished: T4-type, 44RR-type, RB43-type, and RB49-type viruses. The *Spounavirinae *contain the "SPO1-"and "Twort-like viruses."

**Conclusion:**

The hierarchical clustering of these groupings provide biologically significant subdivisions, which are consistent with our previous analysis of the *Podoviridae*.

## Background

We recently described methods aimed at unifying classical and genomic classification of bacteriophages by integration of protein sequence data and physicochemical parameters. We developed two protein sequence similarity-based tools, CoreExtractor and CoreGenes [1], to parse-out and quantify relationships between pairs of phages resulting in a single correlation score [2]. This analysis is followed by a deconstruction and literature analysis of the known morphological and physicochemical characteristics of these phages. The biological interpretation of molecular correlations between 55 fully sequenced *Podoviridae *show that this approach agrees with the current phage classification of the International Committee on Taxonomy of Viruses (ICTV) and suggests that, generally, horizontal gene transfer only partially masks evolutionary relationships between phages. Using a cut-off value of 40% homologous proteins, we verified relationships between phages known to be similar and identified several new bacteriophage genera. At the 20-30% homology level, we identified relationships of a higher order justifying the introduction of the subfamily taxonomical category.

The *Myoviridae *in the VIIIth ICTV Report comprise five genera of bacteriophages (Mu, P1, P2, SPO1, and T4-like viruses) and one genus of archeal viruses, phiH. I3 and phiKZ-like phages have been recently proposed as additional genera http://www.ncbi.nlm.nih.gov/ICTVdb/Ictv/fs_myovi.htm. These genera include only a small fraction of presently known myoviruses with fully sequenced genomes [3]. We analyze and interpret here the correlations between 102 *Myoviridae *genomes found in the National Center for Biotechnology Information (NCBI) and the Tulane University T4 Genome databases.

## Results and discussion

**Figure 1:**
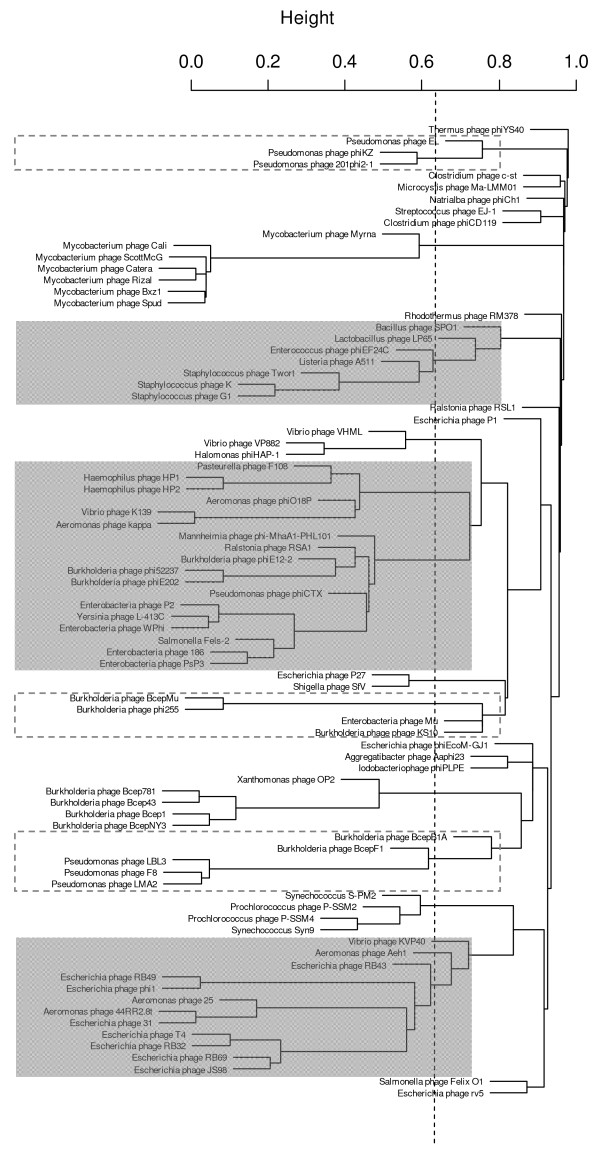
**Hierarchical cluster dendrogram of the analyzed *Myoviridae***. The relative dissimilarity between the phage proteomes (between 0.0 and 1.0) forms the basis for the proposed groupings. The dotted lines reflects the cut-off value used for the establishment of genera, used consistently for all *Myoviridae *and the previously defined *Podoviridae *[107]. Subfamily and tentative subfamily groupings are indicated in the grey and dotted boxes, respectively.

Figure 1 shows the correlation, based on the CoreExtractor distance measure, among all available *Myoviridae *genomes in the NCBI databases. To verify and more subtly compare individual correlations, the CoreGenes approach was applied to subsets of related phages, including several genomes not currently available in public databases (Table 1). As in previous analyses of the *Podoviridae *[2], threshold values of 40% and 20% (and 0.6 and 0.8 relative dissimilarity, respectively) of homologous proteins strongly suggest genus and subfamily boundaries, respectively (Additional file 1). They are corroborated by morphological, molecular or physiological data and discussed in the paragraphs below.

**Table 1:** Comparison of CoreExtractor and CoreGenes and the classification of fully sequenced members of the *Myoviridae*

I. TEEQUATROVIRINAE				
Percent identity				
1. The T4-like viruses		Accession No.	CoreExtractor	CoreGenes
	T4-type phages			
	*Escherichia *phage T4	NC_000866	100	100.0
	*Escherichia *phage JS10	NC_012741	Not determined	72.7
	*Escherichia *phage JS98	NC_010105	77	74.1
	*Escherichia *phage RB14	NC_012638	Not determined	83.5
	*Escherichia *phage RB32	NC_008515	88	84.2
	*Escherichia *phage RB51	NC_012635	Not determined	85.6
	*Escherichia *phage RB69	NC_004928	73	73.4
				
	44RR2.8-type phages			
	*Aeromonas *phage 44RR2.8t	NC_005135	100	100.0
	*Escherichia *phage 31	NC_007022	98	97.6
	*Aeromonas *phage 25	NC_008208	82	82.5
				
	RB43-type phages			
	*Escherichia *phage RB43	NC_007023	100	100.0
	Escherichia phage RB16	Tulane	Not determined	84.2
				
	RB49-type phages			
	*Escherichia *phage RB49	NC_005066	100	100.0
	*Escherichia *phage JSE	NC_012740	Not determined	93.6
	*Escherichia *phage φ1	NC_009821	97	97.1
2. The KVP40-like viruses				
	*Vibrio *phage KVP40	NC_005083	100	100.0
	*Vibrio *phage nt-1	Tulane	Not determined	80.8
				
	*Acinetobacter *phage 133	Tulane	Not determined	39.9
	*Aeromonas *phage Aeh1	NC_005260	28	35.6
	*Aeromonas *phage 65	Tulane	Not determined	34.9

**II PEDUOVIRINAE**				
1. The P2-like viruses				
	*Enterobacteria *phage P2	NC_001895	100	100.0
	*Enterobacteria *phage Wφ	NC_005056	89	90.7
	*Yersinia *phage L-413C	NC_004745	95	88.4
	*Enterobacteria *phage 186	NC_001317	72	74.4
	*Enterobacteria *phage PsP3	NC_005340	70	72.1
	*Salmonella *Fels-2	NC_010463	65	67.4
	*Salmonella *SopEφ	AY319521	Not determined	62.8
	*Burkholderia *phage φE202	NC_009234	51	55.8
	*Mannheimia *phage φ-MhaA1-PHL101	NC_008201	51	55.8
	*Pseudomonas *phage φCTX	NC_003278	53	53.5
	*Burkholderia *phage φ52237	NC_007145	49	51.2
	*Ralstonia *phage RSA1	NC_009382	49	51.2
	*Burkholderia *phage φE12-2	NC_009236	49	48.8
2. The HP1-like viruses				
	*Haemophilus *phage HP1	NC_001697	100	100.0
	*Haemophilus *phage HP2	NC_003315	97	85.7
	*Pasteurella *phage F108	NC_008193	57	59.5
	*Vibrio *phage K139	NC_003313	51	54.8
	*Vibrio *phage κ	NC_010275	49	54.8
	*Aeromonas *phage ΦO18P	NC_009542	44	50.0

**III. SPOUNAVIRINAE**				
1. The SPO1-like viruses				
	*Bacillus *phage SPO1	NC_011421	100	100.0
2. The Twort-like viruses				
	*Staphylococcus *phage Twort	NC_007021	100	100.0
	*Staphylococcus *phage K	NC_005880	74	43.5
	*Staphylococcus *phage G1	NC_007066	97	56.9
	*Listeria *phage P100	NC_007610	51	34.8
	*Listeria *phage A511	NC_009811	51	35.4
	Peripherally related:			
	*Enterococcus *phage φEC24C	NC_009904	32	31.8
	*Lactobacillus *phage LP65	NC_006565	25	26.2

**OTHER ICTV-RECOGNIZED GENERA**				
1. The Mu-like viruses				
	Enterobacteria phage Mu	NC_000929	100	100.0
2. The P1-like viruses				
	*Escherichia *phage P1	NC_005856	100	100.0
	*Escherichia *phage P7	AF503408	Not determined	87.3

**PROPOSED GENERA WITHIN THE *MYOVIRIDAE***				
1. The Bcep781-like viruses				
	*Burkholderia *phage Bcep781	NC_004333	100	100.0
	*Burkholderia *phage Bcep43	NC_005342	98	95.5
	*Burkholderia *phage Bcep1	NC_005263	85	90.9
	*Burkholderia *phage BcepNY3	NC_009604	87	92.4
	*Xanthomonas *phage OP2	NC_007710	52	50.0
2. The BcepMu-like viruses				
	*Burkholderia *phage BcepMu	NC_005882	100	100.0
	*Burkholderia *phage φE255	NC_009237	89	86.8
3. The FelixO1-like viruses				
	*Salmonella *phage Felix O1	NC_005282	100	100.0
	*Escherichia *phage wV8	EU877232	Not determined	92.4
	*Erwinia *phage φEa21-4	NC_011811	Not determined	52.7
4. The HAP1-like viruses				
	*Halomonas *phage φHAP-1	NC_010342	100	100.0
	*Vibrio *phage VP882	NC_009016	Not determined	73.9
5. The Bzx1-like viruses				
	*Mycobacterium *phage Bzx1	NC_004687	100	100.0
	*Mycobacterium *phage Catera	NC_008207	95	95.4
	*Mycobacterium *phage Cali	NC_011271	92	93.6
	*Mycobacterium *phage ScottMcG	NC_011269	93	94.5
	*Mycobacterium *phage Rizal	NC_011272	95	95.9
	*Mycobacterium *phage Spud	NC_011270	97	98.2
	*Mycobacterium *phage Myrna	NC_011273	39	46.3
6. The phiCD119-like viruses				
	*Clostridium *phage ΦCD119	NC_007917	Not determined	100.0
	*Clostridium *phage ΦCD2	NC_009231	Not determined	50.6
	*Clostridium *phage ΦCD27	NC_011398	Not determined	36.7
7. The phiKZ-like viruses				
	*Pseudomonas *phage φKZ	NC_004629	100	100.0
	*Pseudomonas *phage 201φ2-1	NC_010821	50	51.0
Peripherally related:				
	*Pseudomonas *phage EL	NC_007623	30	21.9
8. The PB1-like viruses				
	*Pseudomonas *phage PB1	NC_011810	Not determined	100.0
	*Pseudomonas *phage F8	NC_007810	Not determined	95.7
	*Pseudomonas *phage LBL3	NC_011165	97	89.2
	*Pseudomonas *phage LMA2	NC_011166	97	95.7
	*Pseudomonas *phage SN	NC_011756	Not determined	92.5
	*Pseudomonas *phage 14-1	NC_011703	Not determined	92.5
	*Burkholderia *phage BcepF1	NC_009015	44	43.0
Peripherally related:				
	*Burkholderia *phage BcepB1A	NC_005886	22	24.7

**PRELIMINARY GROUPINGS AND UNRELATED PHAGES**				
(cyanomyoviridae)				
	*Synechococcus *S-PM2	NC_006820	100	100.0
	*Synechococcus *Syn9	NC_008296	41	41.5
	*Prochlorococcus *phage P-SSM2	NC_006883	35	40.3
	*Prochlorococcus *phage P-SSM4	NC_006884	35	39.8
(phage SfV and relatives)				
	*Shigella *phage SfV	NC_003444	100	100.0
	*Escherichia *phage P27	NC_003356	42	43.1
*Aggregatibacter *phage Aaφ23		NC_004827	100	100.0
*Clostridium *phage c-st		NC_007581	100	100.0
*Escherichia *phage rV5		NC_011041	100	100.0
*Escherichia *phage P4		NC_001609	100	100.0
*Escherichia *phage φEcoM-GJ1		NC_010106	100	100.0
Iodobacteriophage phiPLPE		NC_011142	100	100.0
*Lactobacillus *phage Lb338-1		NC_012530	100	100
*Microcystis *phage Ma-LMM01		NC_008562	100	100.0
*Natrialba *phage φCh1		NC_004084	100	100.0
*Ralstonia *phage RSL1		NC_010811	100	100.0
*Rhodothermus *phage RM378		NC_004735	100	100.0
*Streptococcus *phage EJ-1		NC_005294	100	100.0
*Thermus *phage φYS40		NC_008584	100	100.0

### A. *Myoviridae *Subfamilies

#### I. *Teequatrovirinae*

##### 1. T4-like viruses *nova comb*

The ICTV currently lists only six sequenced viruses as members of the T4 phage genus, namely enterobacterial phage T4, *Acinetobacter *phage 133, *Aeromonas *phages Aeh1, 65 and 44RR2.8t, and *Vibrio *phage nt-1. However, the scientific literature and public databases abound with descriptions of "T4-like" phages and the analysis of complete genome sequences indicates that the T4-related phages constitute one of the largest groups of bacterial viruses. This corroborates ecogenomic studies on the diversity of these viruses as apparent in the heterogeneity of capsid (gp23) genes in isolates from Japanese rice fields [4], marine systems [5,6], and from Lithuania [7], Bangladesh and Switzerland [8]. These studies suggest that the fully sequenced T4 phages are but a small fraction of the T4-related genomes in nature. Nevertheless, there are clear commonalities among all sequenced "T4-like" genomes from different host groups, including the cyanophages, namely a set of 33-35 genes that have persisted during the evolution of genomes with sizes from 160 to 250 kb [9]. This core of genes seems to have resisted divergence throughout evolution. Nevertheless, these horizontal substitutions do not erase the evidence of the global relationship between phages and clear hybrid phages within this group have not been identified to date [10,11]. Work done at Tulane University [10,11], led to the tentative conclusion that it takes about 33 T4 genes to determine a genetic program that controls lytic phage development in the host cell.

Based on the *Myoviridae *cluster dendrogram (Figure 1), the current ICTV genus "T4-like viruses" can be subdivided into two genera and several subgroups. By analogy to the T7-related podoviruses, now named the *Autographivirinae*, the former ICTV genus was raised to the rank of a subfamily, the *Teequatrovirinae*, named after the best-studied of these phages, coliphage T4. The first genus, the "T4-like viruses", includes what were previously termed the T-even and "pseudo-T-even" phages [12,13]. Our name perpetuates the old ICTV nomenclature, but is now limited to enterobacterial and *Aeromonas *phages. The KVP40 phages, consisting of two former members of the "schizo-T-evens" [14] form the other genus.

The "T4-like viruses" are morphologically indistinguishable and have moderately elongated heads of about 110 nm in length, 114 nm long tails with a collar, base plates with short spikes, and six long kinked tail fibers. Within this assemblage, we identified four distinct subtypes with >70% protein similarity. These are the T4-type phages (phages T4, JS10, JS98, RB14, RB32, RB51, RB69), 44RR-type (phages 44RR2.8t, 31, 25), RB43-type (RB43, RB16), and the RB49-type viruses (RB49, JSE, φ1). They can be subdivided by the presence of specific encoded proteins as outlined in Table 2. In the subtype T4 phages, three specific proteins with defined functions (Pin, MotB, ModA) were found. Pin is an inhibitor of the host's Lon protease [15,16], while the other two proteins function to modulate transcription [17,18].

**Table 2:** Type-specific proteins in T4 phages

Type (host)	Genome size (in kb)	Type-specific proteins
T4 (*E. coli*)	165.9-170.5	NP_049650, 049704, 049747, 049694 (Pin), 049626 (MotB), 049635 (ModA)
44RR2.8t (*Aeromonas*)	161.5-173.6	NP_932430, 932451, 932460, 932567, 932569, 932577
RB49 (*E. coli*)	164.1	NP_891619, 891621, 891622, 891626, 891736, 891753, 891760, 891800, 891816
RB43 (*E. coli*)	178.7	YP_239033, 239034, 239054, 239086, 239094, 239097, 239130, 239215, 239216, 239241

Heteroduplex analyses indicate that coliphages T2, T4 and T6 share >85% sequence similarity [19], warranting their inclusion, in spite of lack of detailed sequence data for T2 and T6, into the T4-type subgroup. The DNA of the T-even phages contains 5-hydroxymethylcytosine (5-HMC). While this modified nucleotide is common in T4-related phages [20], its presence has not been ascertained biochemically in the other phages (JS98, RB14, RB32, RB69) included in this subgroup. T4 gp*42 *dCMP hydroxymethylase and Alc that blocks transcription from cytosine containing DNA are required for the incorporation of 5-HMC rather than cytosine into T-even DNA. Genes specifying homologs of the T4 gp*42 *and Alc proteins are also present in the 44RR2.8t-type phages.

##### 2. KVP40-like viruses

The KVP40 viruses comprise two marine vibriophages, KVP40 and nt-1, with genomes of approximately 246 kb. KVP40 infects *Vibrio parahaemolytius *and was isolated from seawater. Phage nt-1 infects *Vibrio natriegens *and originates from a coastal marsh. The phages differ from T4 in head length (137 nm vs. 111 nm), but are identical to phage T4 in tail morphology. KVP40 has a feather of decoration proteins on its head [21,22].

Three other T4 phages do not fit into these groups: *Acinetobacter *phage 133, *Aeromonas hydrophila *phage Aeh1 and *Aeromonas salmonicida *phage 65. Morphologically, phage 133 is identical to T4, whereas Aeh1 and 65 have the same heads of 133 nm in length as *Vibrio *phages KVP40 and nt-1. They were considered to be part of the "schizo-T-even" group [23] and have a T4-type tail structure [20]. CoreGenes and our supplementary phylogenetical analyses indicate that these phages are too dissimilar, by our criteria, to be included into one of the genera listed above.

The four marine cyanophages (P-SSM2, P-SSM4, S-PM2 and Syn9) infect *Synechococcus *or *Prochlorococcus *strains and harbor T4 genes causing this group to be named the "exo-T-evens" [24,25]. These phages have isometric heads and much longer tails than T4. CoreGenes analysis indicates that they form a group sharing >40% proteins in common. While P-SSM2, P-SSM4 and Syn9 share 93 proteins, they show considerable dissimilarity in appearance, size, and DNA content (Table 3). Phylogenetic analysis based upon sequence alignments of gp*20 *(portal vertex protein [26]) and photosystem II protein D1 [27,28] indicate considerable diversity exist among cultured and environmental cyanophages. This is also confirmed by an analysis of data from the marine virome from the Sorcerer II Global Ocean Sampling expedition [29]. Based upon these observations, we feel that the creation of genera within cyanophage myoviruses is premature at the present time.

**Table 3:** T4 cyanophages

Phage	Head, nm	Tail length, nm	DNA size, kb	ORFs	References
P-SSM2	110*	100*	252	327	[103]
P-SSM4	70*	200*	178	198	[103]
S-PM2	67	200	187	239	[104,105]
Syn9	87	150	173	226	[106]

*From published micrographs.

*Rhodothermus marinus *phage RM378 (NC_004735) is a virus said to have a head of 95 × 85 nm and a tail of 150 nm in length [30]. It was called a "ThermoT-even phage" by Filée et al. [6], but our CoreGenes analysis reveals that its proteins shows minimal sequence similarity to any T4-related virus.

#### II. Peduovirinae

This subfamily is a large phage group derived from the ICTV genus "P2-like phages" and is named the *Peduovirinae*. Virions have heads of 60 nm in diameter and tails of 135 × 18 nm. Phages are easily identified because contracted sheaths tend to slide off the tail core. The subfamily falls into three different groups. As shown by CoreExtractor and CoreGenes analyses, and using the 40% similarity criterion for inclusion into the same genus, phage HP1 has only 9 genes in common P2. Even if other P2 phages are considered, HP1 shares only 17 genes with any phage of the "P2-like" genus. Using the 40% similarity criterion for inclusion into the same genus, it is therefore justified to consider P2 and HP1 as members of different genera and to upgrade the present genus "P2 phages" to a subfamily.

##### 1. P2-like viruses *nova comb*

This genus includes P2 itself and its extensively studied relative, coliphage 186. Both originate from the Pasteur Institute in Paris, France. Phage P2 is one of three phages (P1, P2, P3) isolated by G. Bertani in the beginning of the 1950's from the "Li" (Lisbonne and Carrère) strain of *E. coli *[31]. Later on, F. Jacob and E. Wollman isolated phage 186 and many other viruses from enterobacteria collected by L. Le Minor [32]. The reason for the early interest in these phages was that P2 and 186 are temperate. The analysis of the genetic control of these two modes was the starting point for ongoing fertile research on phage biology and molecular biology in general.

The genomes of phage P2 and 186 were the first P2 genomes to be fully sequenced and analyzed. Almost all P2 and 186 genes have been assigned a function [33-35]. Coliphages WΦ and L-413C are very similar to P2 in both gene content and gene order. They are closely related to each other, sharing all but one protein. The only genes of these phages that differ from P2 are the lysogeny-related genes, which may have been horizontally acquired and are totally different, but have been inserted at the same locations into all genomes. The only exception to this is that phage P2 has a 786 bp ORF (orf30) with unknown function inserted between the *S *and *V *genes. There is no such insertion in WΦ and L-413C, but *Pseudomonas *phage ΦCTX (see below) has another uncharacterized ORF located at this position. Enterobacterial phages 186, PSP3, Fels-2, and SopEΦ also share their overall gene order and many genes with P2, but the genes are more diverged. Unlike P2, these phages are UV-inducible due to the presence of the *tum *gene. In addition, they have a different lysis-lysogeny switch region. P2 phages seem to have either of two different proteins for repression of the lytic cycle. P2, WΦ and L-413C have the repressor gene *C *whereas 186, PSP3, Fels-2, SopEΦ, HP1, HP2, and K139 (below) instead have the sequence-unrelated genes *CI *and *CII*, both of which are equally needed for establishing lysogeny.

*Mannheimia *phage Φ-MhaA1-PHL101, *Pseudomonas *phageΦCTX, and *Ralstonia *phage RSA1 have many P2 genes and an overall order of structural genes that is P2-like, although interspersed with some uncharacterized genes. Their presumed regulatory gene regions include additional putative and uncharacterized ORFs. Phage ΦCTX has only the P2 regulatory gene *ogr *(transcriptional activator of the late genes) and the recombination enzyme *int *(integrase), Φ-MhaA1-PHL101 has repressor (CI) and antirepressor (Cro) equivalents which are most closely related to the regulatory proteins of the P22-like enterobacteria phage ST104 than to P2.

Phage RSA1 seems to have only one P2-related regulatory gene, the *ogr *gene, although it is more related to the Ogr/Delta-like gene in ΦCTX. The RSA1 integrase is more similar to the integrases of the P2-like *Burkholderia *phages (ΦE202, Φ52237, and ΦE12-2 and P22-like viruses.

##### 2. HP1-like viruses

The genome architecture of HP1 [36] and its close relative, HP2, resembles that of P2 although their *cos *sites, as with *Pseudomonas *ΦCTX [37], are located next to *attP *rather than downstream of the portal protein-encoding gene as it is in P2. The P2 gene order is also conserved in *Vibrio *phages K139 [38] and κ and the *Pasteurella *phage F108 [39]. As in P2, the genomes can be divided into blocks of structural and regulatory genes. The structural genes are more similar in HP1 and HP2 than the regulatory genes. The six genes coding for capsid proteins are arranged in the same order in HP1 phages and many P2 phages. The other structural genes, coding mainly for tail components, show generally no similarity to those of P2 phages. Only some of the regulatory genes are similar in both HP1 and P2 phages, e.g., *int*, *CI*, and *repA*. Regulatory genes in general are more conserved within the HP1 group.

*Aeromonas *phage ΦO18P [40] is included into the HP1 phages. It contains slightly more genes related to HP1 than to P2, although, when looking at individual proteins, it sometimes appears to have an intermediate position. Its Rep protein is very similar to the DNA replication protein of *Salmonella *phage PSP3 and the A protein of phages K139, F108, WΦ, and P2 homologs. The ΦO18P major capsid protein is similar to the capsid proteins of phages K139, ΦCTX, 186, and the *Burkholderia *phages.

#### III. The *Spounavirinae*

This proposed subfamily contains the ICTV-recognized genus "SPO1-like viruses" and, on the basis of our results, a proposed new genus (the "Twort-like viruses") and two peripherally related viruses, *Lactobacillus plantarum *phage LP65 [41] and *Enterococcus faecalis *phage φEF24C [42,43]. All of these are virulent, broad-host range phages which infect members of the *Firmicutes*. They possess isometric heads of 87-94 nm in diameter and conspicuous capsomers, striated 140-219 nm long tails, a double base plate, and globular structures at the tail tip. The latter have been resolved as base plate spikes and short kinked tail fibers with six-fold symmetry [44]. Members of this group usually possess large (127-142 kb) nonpermuted genomes with 3.1-20 kb terminal redundancies [45,46]. The proposed name for this subfamily is derived from SPO plus *una *(latin for "one").

While the head diameter of *Bacillus *phage SPO1, of 87 nm [47], is consistent with membership in the group, its tail is significantly shorter than that of most members (140-150 nm) [3,48], and, the DNA contains 5-hydroxymethyluracil (HMU) rather than thymine. The outliers of this group comprise phages LP65 [41] and φEF24C [42,43]. At 193 nm, the tail of phage LP65 is similar in length to that of other members of this group, but its genome is not terminally redundant [41]. Lastly, the genome size (142 kb), proteome and morphology of *Enterococcus *phage φEF24C is clearly consistent with membership in this group (head diameter 93 nm; tail length 204 nm), but its genome is circularly permuted. Their close relationship was discussed in a recent paper [44].

Using a BLASTP raw threshold score of 100 and CoreGenes 3.0 http://binf.gmu.edu:8080/CoreGenes3.0/ to compare the proteomes of Twort, A511, LP65, and φEF24C against SPO1, we identified two clusters of genes which are conserved. These corresponded to packaging and morphogenesis genes (SPO1 gp*2.11 *to gp*16.2*); and the cluster of replication genes, including helicase, exonuclease, primase, and resolvase (SPO1 gp*19.5 *- gp*24.1*). The DNA polymerases (SPO1 gp*31 *and homologs) of these phages are related more closely to bacterial-type I DNA polymerases than other phage deoxynucleotide polymerizing enzymes. The presence of host-related proteins in viruses has been observed by Dinsdale et al. [49] and elegantly explained by Serwer [50]. Metagenomic studies by the former group indicate the presence of numerous host-related proteins, including those related to motility and chemotaxis, in the virome fractions. While the functional significance of photosynthetic protein *psbA *in cyanophage genomes has been conclusively shown [51,52], the presence of host-related sequences should still be considered with healthy skepticism if the only data is the presence of homologs.

##### 1. SPO1-like viruses

The current ICTV genus "SPO1 viruses" comprises some 10 *Bacillus *phages and *Lactobacillus *phage 222a; only the genome of SPO1 has been sequenced [53]. All SPO1-like *Bacillus *phage genomes that have been studied contain 5-hydroxymethyluracil (HMU) instead of thymine and encode dUMP hydroxymethylase activity (SPO1 gp*29*). This phage also contains the unique 171-amino acid head decoration protein gp*29.2*. Whether this is unique to members of this genus will require the sequencing of additional genomes. Using cryo-electron microscopy, Duda and coworkers [54] confirmed the earlier observation [47] that the icosahedral head of SPO1 head has the triangulation number T = 16 rather than the more common T = 25. This feature is also shared with eukaryotic herpesviruses.

##### 2. Twort-like viruses

The phages form a fairly homogeneous group of virulent phages infecting staphylococci (Twort, G1, K) [55] and *Listeria *(A511, P100) [56]. The group is named after phage "Twort," which may be a descendant of the original bacteriophage described by F.W. Twort in 1915 [57]. Apparently, this phage was deposited at the Pasteur Institute of Paris in 1947 when Twort was invited there to retell the story of his discovery (personal communication to H.-W.A. by J.-F. Vieu, curator of the phage collection of the Pasteur Institute; 1983).

### B. Additional ICTV-recognized genera

#### 1. Mu-like viruses

Phage Mu is morphologically almost identical to phage P2. Although phage Mu shares features (e.g. replicative transposition) with BcepMu [58] and two siphoviruses, *Pseudomonas *phages B3 and D3112 [59,60], this phage holds a unique position within the *Myoviridae*, since its proteome displays only limited homology to any other completely sequenced phage genome.

Mu and P2 have only 4 proteins in common (overall 9.8% similarity). P2 differs from Mu by genome size (33.6 kb vs. 36.7 kp in Mu), the number of proteins (43 proteins vs. 55 in Mu), gene order, and the presence of a single capsid protein and cohesive ends in its DNA. By contrast, Mu has two capsid proteins and two sets of tail fiber genes and replicates via transposition, which is a very rare mode of replication. Mu shares this characteristic with BcepMu, but BcepMu has no tail fiber inversion system and only a limited proteomic correlation to Mu (9 gene homologs; 16.4% similarity).

Only coliphage D108, as shown by heteroduplex analysis, shows significant similarity to Mu to warrant inclusion in the Mu genus [61]. Unfortunately, only portions of the genome of D108 have been sequenced. Putative Mu proviruses have been reported in a wide range of bacteria [62-64]. CoreGenes analysis revealed that only some of them can be reasonably described as Mu proviruses, namely, *Escherichia blattae *prophage MuEb [65], *Haemophilus influenzae *Rd prophage Hin-Mu [66], and *Shewanella oneidensis *prophage MuSo2 [NC_004347].

#### 2. P1-like viruses

This small genus comprises coliphage P1, famous for its cre recombinase and the large-insert cloning vectors engineered on the basis of the phage genome [67], P7 (AF503408) and its not yet sequenced putative relative D7. Phage P1 stands out from any of the phages described here by its morphology. Phage P1 differs from the phages described here by size and morphology. It has a very large head of approximately 85 nm in diameter and a very long tail of 228 × 18 nm in the extended state. Tails have base plates and 90 nm long, kinked fibers. The tails of related, not yet sequenced phages of enterobacteria and *Aeromonas *vary between 170 and 240 nm in length. All phages of this group produce three types of head-size variants (small, normal, intermediate).

### C. Additional genera within the *Myoviridae*

#### 1. Bcep781-like viruses

"Bcep" stands for ***B****urkholderia ****cep****acia*, and phages with this designation infect bacteria belonging to the *B. cepacia *genomic complex. The Bcep781 phages form a group of virulent myophages of which the genome sequence of five members, Bcep781, Bcep1, Bcep43, BcepNY3 and *Xanthomonas *phage OP2, is known [68,69]. The Bcep781 phages are small viruses with distinctly shorter tails than P2, Mu, and BcepMu [68].

The genomes of these phages range from 46 to 49 kb in size and encode 66 to 71 proteins. The four Bcep phages encode a single tRNA each. They form a homogeneous phage group not just in terms of sequence, but also by their distinctive genome organization compared to other groups. The genomes of the Bcep781 phages are divided into four gene clusters encoded on alternate strands such that, using Bcep781 as the example, genes *1 *through *19 *and *29 *through *51 *are present on the bottom strand while genes *20 *through *28 *and *52 *through *66 *are present on the top strand. Head genes are located in the first cluster and tail genes are located in the third cluster. The virion major capsid and decoration proteins, Bcep781 gp*12 *and gp*13*, were identified by protein sequencing and show some similarity to head proteins from the "PB1-like viruses" group. Several tail morphogenesis proteins, corresponding to Bcep781 gp*29 *through gp*52*, can be linked to P2 tail genes by PSI-BLAST. In contrast to structural genes, genes for DNA replication and lysis are scattered throughout the genome. The lysis genes of these phages are not organized into a cassette but instead overlapping Rz and Rz1 genes are separated from the endolysin and holin genes [70]. A distinctive feature of these phages is the presence of highly, maybe completely, circularly permuted genomes. The terminases of these phages are strongly related to other *pac-*type phages that also have highly permuted genomes [71].

#### 2. BcepMu-like viruses

This group was named "BcepMu-like viruses" because, like Mu and unlike most other phages, its members utilize transposition for replication. The distinctive genomic feature implicating the use of replicative transposition is the presence of random host DNA sequences at either end of the packaged virion DNA [58]. These host sequences are derived from excision of prophage DNA from random sites scattered over the host genome. This requires fundamental differences in terminase function as compared to more typical terminases that utilize concatemers of phage genomic DNA as a substrate. This is reflected by the homology between BcepMu TerL and Mu TerL. Another genome feature shared by BcepMu and Mu is the presence of genomic terminal CA dinucleotide repeats, a feature common in many transposons. Furthermore, BcepMu and Mu seem to be morphologically identical.

Despite these similarities, BcepMu and its close relative φE255 have marked differences in genome organization and minimal overall protein sequence similarity to Mu, explaining why they have not been grouped together. The putative BcepMu transposase is not related to the Mu transposase, TnpA, but instead is a distant member of the Tn552-IS1604 transposase family. The BcepMu genome is organized into two clusters, with genes *1 *through *13 *encoded on the bottom strand and genes *17 *through *52 *on the top strand. The cluster of bottom strand genes includes transcription regulators, the transposase, and a number of small genes of unknown function. The lysogeny control region is likely to include genes *16 *and *17*, located at the interface of the bottom strand/top strand gene clusters. This is followed by a lysis cassette consisting genes encoding a holin, endolysin, Rz and Rz1. Proteins *27 *through *51 *encompass the head and tail morphogenesis cassette. The BcepMu tail biosynthetic cassette proteins are recognizably related both in sequence and in gene order to those of coliphage P2.

BcepMu is present as a prophage in many *B. cenocepacia *strains of the human pathogenic ET2 lineage [58,72]. Phage φE255 is a phage of the soil saprophyte *B. thailandensis *[NC_009237]. BcepMu phages, however, are not limited to *Burkholderia *hosts as related prophage elements have been identified in the genomic sequence of many other bacteria, for example *Chromobacterium violaceum *[NP_901809].

#### 3. Felix O1-like viruses

*Salmonella *phage Felix O1 has a relatively large head (70 nm in diameter) and a tail of 138 × 18 nm characterized by subunits overlapping each other like roof tiles and showing a criss-cross pattern like phages PB-1 and F8. Notably, it exhibits small collars and eight straight tail fibers. Upon contraction, the base plate separates from the sheath. The type virus Felix O1 is widely known as a diagnostic *Salmonella-*specific phage [21]. Until recently, the genomic sequence (86.1 kb) of phage Felix O1 was unique and was considered, as such, a "genomic orphan", but two related genomes have been recently characterized, though their sequences have yet to be deposited to the public databases. They are coliphage wV8 and *Erwinia amylovora *phage φEa21-4 (DNA sizes 88.5 and 84.6 kb, respectively [73,74].

#### 4. HAP1-like viruses

This genus contains two marine phages, *Vibrio parahaemolyticus *phage VP882 (NC_009016) and *Halomonas aquamarina *phage φHAP-1 [75]. Both are temperate viruses possessing 38-43 kb genomes which lack integrase genes. While our proteomic analysis and the literature suggests that *Vibrio harveyi *phage VHML [76,77] should be included in this genus, there is no evidence that this phage can be propagated: it is only produced after induction, does not plaque, and must be considered a defective prophage. The data presented by Mobberley et al. [78] show that φHAP-1 exists as a linear prophage in lysogens and possesses a protelomerase (ORF34, YP_001686770.1) and a partitioning protein (ParA homolog, ORF33, YP_001686769.1) which are homologous to proteins encoded by VHML and VP882. While these viruses share some homology with the coliphage P2, this is largely restricted to the genes associated with tail morphogenesis V (gp*V, W, J, I, H, G*) and F operons (gp*FI, FII, E, T, U, D*). Based upon their radically different life cycle from the other P2 phages, we have chosen not to include them in the *Peduovirinae*.

#### 5. Bzx1-like or I3-like viruses

Myoviruses are exquisitely rare in the *Actinobacteria *(only an estimated 1% of all attempts to isolate phages from cultures was successful [79]). Phages I3, Bzx1 and Catera are characterized by heads of 80 nm in diameter and unusually short tails of 80 nm in length with a cup-shaped base plate. They do not resemble any other mycobacteriophages nor any other myovirus. We propose that this genus contains the following eight *Mycobacterium smegmatis *bacteriophages: I3, Bxz1, Cali, Catera, Myrna, Rizal, ScottMcG and Spud. Phage I3, which has been the first to be described, is the type virus of the newly proposed myovirus genus although it has not yet been fully sequenced. Within this assemblage, we identified a distinct subtype which show >90% protein similarity to Bxz1 (Cali, Catera, Rizal, ScottMcG and Spud) and genomes of 154-156 kb [80,81]. Mycobacteriophage Myrna, with a genome of 164 kb, shares approximately 45% of proteins with the Bxz1 subgroup phages. Interesting features include the presence of adenylosuccinate synthase homologs among the Bxz1 subgroup (gp*250*) and its absence in the genome of Myrna. The latter possesses several proteins not present in the Bxz1 group, including the large hypothetical proteins gp*187 *(YP_002225066.1) and gp*243 *(YP_002225120.1), a putative nicotinate phosphoribosyltransferase (gp*263*, YP_002225140.1) and ATP-dependent protease (gp*262*, YP_002225139.1).

#### 6. phiCD119-like viruses

These are all integrative temperate phages of *Clostridium difficile *with genomes ranging from 51-60 kb in size and a mol%G+C of 28.7-29.4 [82-84]. The genus is named after its first fully sequenced member. In each case, the electron micrographs are of poor quality [84,85] or the measurements are very variable with large standard deviations [85]. Virus head diameters are given as 50-65 nm and tail lengths are said to range from 110 to 210 nm [82-84]. In certain cases, their annotation is also questionable, The multiple repressor/antirepressors annotated in the genomes of ΦCD27 and φC2 do not appear to contain helix-turn-helix or other DNA binding motifs [86]; nor the presence, in the latter phage, of ParA/ParB homologs. What unites these viruses, in addition to similar proteomes, is the presence in each of a cytosine-C5 specific DNA methylase (pfam00145, DNA_methylase, C-5 cytosine-specific DNA methylase; ΦCD119 protein YP_529611.1) and a DNA replication cassette composed of three proteins: a DnaD (primosome recruiting protein, presumably analogous to lambda gp*O *and P22 gp*18*; ΦCD119 protein YP_529603.1), a hypothetical protein (misidentified in ΦCD27 as a putative resolvase/integrase and missed entirely in the annotation of ΦCD119) and a single-stranded DNA binding protein.

#### 7. phiKZ-like viruses

Phages φKZ and EL are members of a group of giant phages isolated, to date, only in *Pseudomonas *species. Their heads are isometric, 120 nm in diameter, and they possess 190 nm-long tails. The phage heads contain an inner body. The DNA of φKZ is over 280 kb in size and has 306 ORFs, most of which are unrelated to ORFS of any known protein [87], while EL contains 201 ORFs within its 211 kb genome [88]. These two phages and *Pseudomonas *phage Lin68 have recently been proposed as part of a genus "phiKZ viruses" [89]. We now consider that the differences (number of ORFs, mol%G+C, protein homologs) between φKZ and EL exclude EL from membership in the same genus. Indeed, the recent analysis of novel *Pseudomonas *phage 201φ2-1 [90] showed this phage to have a strong correlation to φKZ (167 similar proteins), suggesting that it is a true member of the phiKZ virus genus.

#### 8. PB1-like viruses

This genus is named after the first isolated member of this group (PB1) [91]. Morphological and DNA-DNA hybridization studies by V. Krylov indicated that the following *Pseudomonas *phages were related: E79, 16, 109, 352, 1214, FS, 71, 337, φC17, SL2, B17 [92]. The sequences of a number of viruses belonging to this genus, namely F8, BcepF1, PB1, 14-1, LBL3, LMA2, and SN (P.-J. Ceyssens, personal communication) have now been completed. None of these phages encodes a recognizable integrase, suggesting that they are virulent.

Phage F8 is one of the *Pseudomonas *typing phages from the Lindberg set which includes six more similar phages [93,94]. It possesses a 70-nm wide head with visible capsomers and a 138 nm-long tail, four short straight tail fibers and a base plate that separates from the sheath upon contraction. The tail exhibits no transverse striations, but presents a criss-cross pattern [95]. This criss-cross pattern is a rare feature that has only been observed in phage Felix O1.

BcepF1 was isolated from soil by enrichment culture [96] using a *Burkholderia ambifaria *strain as its host (E.J. Summer and C.F. Gonzalez, unpublished). The BcepF1 genome is 72 kb in size and encodes 127 proteins while the genome of F8 is 66 kb and encodes 91 proteins [97]. Both genomes are organized into four alternating, unequal gene clusters on the top and bottom strands. The phages share 43 recognizable homologous proteins. The shared proteins specify virion morphogenesis, DNA metabolism and packaging and include a number of hypothetical proteins of unknown function.

A striking feature of both F8 and BcepF1 is the large number of small genes, all encoding hypothetical proteins and clustered together. In BcepF1, the first 20 kb of the genome, encoding 62 proteins, is devoted almost exclusively to these. In F8, there are two clusters of 8 kb (encoding gp*1 *through gp*16*, except gp*4*, TerL) and 4 kb (encoding proteins gp*77 *through gp*91*) of primarily small hypothetical novel genes. These heterogeneous regions are largely responsible for the difference in genome size and protein content between the two phages. It has generally been assumed that these small proteins are involved in host take-over (E. Kutter, personal communications) which appears to be substantiated by the results of Liu and coworkers [98].

Phages F8 and BcepF1 have some similarity to myophage BcepB1A, which is itself related in a mosaic fashion to the Bcep781 group of phages [68]; however, these similarities are essentially limited to morphogenetic proteins. As in the Bcep781 phages, several putative tail assembly proteins of F8 and BcepF1 can be linked to those of P2 by PSI-BLAST.

### C. Single phages

In addition to the phage groups listed above, complete genome sequences are available for phages without apparent relatives, namely *Aggregatibacter *(formerly *Actinobacillus*) phage Aaφ23; *Bacillus thuringiensis *phage 0305φ8-36, *Clostridium *phages c-st, *Escherichia *phages φEcoM-GJ1 and rV5; *Microcystis *phage Ma-LMM01, *Ralstonia *phage RSL1, *Rhodothermus *phage RM378; *Streptococcus *phage EJ-1, and *Thermus *phage φYS40. References to these phages may be found in the NCBI RefSeq database.

## General summary

The comparison of proteomes by CoreGenes/CoreExtractor BLASTP programs appears to be a decisive progress in classifying tailed bacteriophages, i.e., our results corroborate the existing ICTV classification of the *Myoviridae *and are generally well compatible with other informatics-based studies (Table 4), like the reticulate clustering based on gene families [99] (Lima-Mendez, personal communication). Our studies also refine certain relationships and suggest new ones. Specifically, we propose three new subfamilies (*Peduovirinae, Teequatrovirinae, Spounavirinae*) and eight new genera (Bcep781, BcepMu, Bzx1, Felix, HAP1, PB1, phiCD119 and phiKZ-like viruses). The individualization of genera containing two or three members as well as of genomic orphans, e.g. coliphage P1 without apparent homologs, is taxonomically as valuable and important as the confirmation of the large T4 and P2 groups and in total agreement with previous informatics-based classifications (Table 4). Our studies once again prove the utility of the dual CoreGenes/CoreExtractor approach to defining relationships between large numbers of virus genomes. These relationships carry evolutionary relevance, since our proteomic analyses, combined with the phylogenetic studies [100], suggest that the *Myoviridae *are mainly influenced by vertical evolution rather than by horizontal gene transfer. As observed in the Cluster dendrogram, the clusters are populated unevenly - several include only one phage while two, the largest, include dozens phages. This reflects the fact that past phage research has focused on coliphages, and suggests that we should broaden our research to include phages from a broader range of bacteria.

**Table 4:** Concordance of classifications

Classification	ICTV	Proteomic Tree 2	----	Phage_Finder	This work
**Reference**	**ICTV VIIIth Report, 2005**	**Edwards and Rohwer, 2005**	**Serwer et al., 2004**	**Fouts, 2006**	

**Approach**	**Traditional**	**Signature genes**	**Large terminase**		**CoreGenes**

Phage or phage group	**T4**, Aeh1, KVP40, RB43, RB49, 25, 31 44RR2.8t, 65	T4	T4, KVP40, RB49		T4, Aeh1, KVP40, RB43, RB49, 25, 31 44RR2.8t, 65
	**P1**			P1	P1
	**P2**, Fels-2, HP1, HP2, K139, φCTX, 186	P2. HP1, HP2, φCTX	P2, Fels-2, HP1, HP2, L413-C, 186; Mu	P2, φCTX, 186 (HP1 occupies a separate position)	P2, Fels-2, HP1, HP2, K139, L-413C, φCTX, 186
	**Mu**	Mu			Mu
	**SPO1**	K		P100, Twort	SPOl, K, P100, Twort
	**ΦH**				

Comparison of our results with those of the ICTV (ICTV VIIIth Report, 2005), Proteomic Tree 2 (Edwards and Rohwer, 2005), Phage_Finder (Fouts, 2006) and phylogeny of terminases (Serwer et al., 2004).

Among the 102 analyzed *Myoviridae*, phage Mu displayed the most significant evidence of horizontal gene exchange. This virus is related to three members of pilus-specific *Siphoviridae *infecting *Pseudomonas aeruginosa *(DMS3, D3112, B3 [59,60,101]), sharing 20 to 40% of its genes with each of them. These phages can be viewed as true hybrids, produced by recombination of different ancestors and, like the couple lambda/P22 (to be described in a future paper), cross family boundaries based on tail morphology. Nonetheless, the majority of *Myoviridae*, when forced to cluster, do so in a logical manner: upgrading of the ICTV genus "P2 phages" to the *Pduovirinae *with two genera ("P2 viruses" and "HP1 viruses") is a straightforward proposal and the same is true for the *Spounavirinae *(SPO1 viruses and Twort viruses).

Relationships among T4-like phages are more complicated. We reject the postulated inclusion of the cyanophages since their overall similarity to T4 is too low for consideration, at least according to our criteria. Comeau and Krisch [29] have recently recognized three groups of T4-related phages. The "Near T4" group containing the T-evens, Pseudo T-evens, and Schizo T-evens; the "Far T4" clade including Exo-T4 phage RM378; and, the "Cyano T4" assemblage. We believe that the latter are sufficiently different from the other T4 viruses to be excluded from the Teequatrovirinae at this time. This implies that this subfamily currently contains two distinct genera: T4 and KVP40 viruses. Within our restricted "T4 phages" genus, four subtypes were identified (T4-type, 44RR2.8t-type, RB43-type and the RB49-type viruses). This is confirmed by the phylogenetic studies of Filée et al. [5] and our unpublished results. Since these subtypes include different species, no equivalent taxonomic level is currently available in the official ICTV classification. Perhaps the introduction of a "subgenus" level should be considered in order to account for the complexity of T4-related phages. Alternately, a general elevation of all taxonomic levels (from the subfamily level) may be envisioned.

This study illustrates the great diversity and biological richness of tailed phages. The number of independent genera is not surprising in view of the antiquity of tailed bacteriophages, which are found in archaea and bacteria and may predate the separation of these domains. It can be expected that many more phage groups will be found or individualized in the future. For example, this study does not include giant *Bacillus *phage G, the largest bacterial virus with a genome of 497,513 bp and 684 genes [102] whose sequence is not yet available for comparison.

We reiterate our statement in our publication on the taxonomy of the *Podoviridae*, "We highly recommend that the entire genome of any newly sequenced phage be thoroughly screened (BLASTX) against the Entrez Query "Viruses [ORGN]" databases to reveal all similarities for quick identification of potential relationships. A validation step using CoreGenes is essential and more precise for individual comparisons [2]."

## Conclusion

*Myoviridae *can be classified by their proteomes into subfamilies and genera. This classification is in close agreement with ICTV - and other informatics-based classifications.

## Methods

### Phages and bioinformatic tools

This study is limited to the genomes of completely sequenced, viable *Myoviridae *from the databases of NCBI http://www.ncbi.nlm.nih.gov/ and the Tulane University at New Orleans, LA (GT4P, "Genomes of the T4 Phages"; http://phage.bioc.tulane.edu/, excluding prophages without a virion stage. We follow here the ICTV which classifies viable viruses only. Prophages and proviruses, prophage fragments, defective viruses, phage-like "bacteriocins", virus-like or phage-likes particles from sections or the environment, viroids, satellite viruses, plasmids, or transposons, or artificial virus hybrids are not considered. CoreExtractor and CoreGenes software were used as described previously [2]. In the case of CoreExtractor, the BLASTX analysis of phage gene products was performed using the NCBI Batch BLAST server, http://greengene.uml.edu/programs/NCBI_Blast.html hosted by the University of Massachusetts at Lowell, MA. Searches were performed against the NCBI nonredundant database (BLOSUM45 matrix, with a 0.05 expectancy cut-off value) (Additional Figure 2). Several versions of CoreGenes are available, with each upgrade incorporating previous functions, at http://www.binf.gmu.edu/genometools.html. In particular, for the current study, a version, CoreGenes3.0beta, was developed specifically for tallying the total number of genes contained in the genomes. It also displays a percent value of genes in common with a specific genome. Additionally, this version finds unique genes between two genomes. The BLASTP stringency setting was set at its default value (75). Proteins containing at least 132 amino acid residues were subjected to BLASTP analysis at NCBI or Tulane University.

### Hierarchical cluster dendrogram

Cluster analysis was used to visualize the structure of the proteomic data. We constructed a dissimilarity matrix from the CoreExtractor matrix. The dissimilarity between two phage genomes was taken as one (1) minus the average of the two reciprocal correlation scores in the CoreExtractor matrix (Figure S1B). Subsequently, single linkage hierarchical clustering was performed using "The R Project for Statistical Computing" software http://www.r-project.org/.

## Competing interests

The authors declare that they have no competing interests.

## Authors' contributions

All the authors contributed to the writing of this manuscript. RL and AMK planned and executed the comparisons. RL, PM and DS developed the software used. Cluster dendrograms were generated by PD.

## Supplementary Material

Additional file 1 **CoreExtractor comparison of *Myoviridae *phages**. A. This Excel figure shows relative correlation scores (above 10%), based on the number of homologous proteins between two phages. Colour tags are added to visualize these correlations (from green to red for increasing correlation scores). B. Corresponding dissimilarity matrix. Click here for file
